# Long-Term Evaluation of Abnormal Behavior in Adult Ex-laboratory Chimpanzees (*Pan troglodytes*) Following Re-socialization

**DOI:** 10.3390/bs3010099

**Published:** 2013-01-31

**Authors:** Elfriede Kalcher-Sommersguter, Cornelia Franz-Schaider, Signe Preuschoft, Karl Crailsheim

**Affiliations:** 1 Institute of Zoology, Karl-Franzens-University of Graz, Universitaetsplatz 2, 8010 Graz, Austria; E-Mails: cornelia.franz@uni-graz.at (C.F.-S.); karl.crailsheim@uni-graz.at (K.C.); 2 Competence Center Apes, Four Paws, Linke Wienzeile 236, 1150 Vienna, Austria; E-Mail: Signe.Preuschoft@vier-pfoten.org

**Keywords:** chimpanzees, abnormal behavior, social deprivation, re-socialization

## Abstract

Adverse rearing conditions are considered a major factor in the development of abnormal behavior. We investigated the overall levels, the prevalence and the diversity of abnormal behavior of 18 adult former laboratory chimpanzees, who spent about 20 years single caged, over a two-year period following re-socialization. According to the onset of deprivation, the individuals were classified as early deprived (EDs, mean: 1.2 years) or late deprived (LDs, mean: 3.6 years). The results are based on 187.5 hours of scan sampling distributed over three sample periods: subsequent to re-socialization and during the first and second year of group-living. While the overall levels and the diversity of abnormal behavior remained stable over time in this study population, the amplifying effects of age at onset of deprivation became apparent as the overall levels of abnormal behavior of EDs were far above those of LDs in the first and second year of group-living, but not immediately after re-socialization. The most prevalent abnormal behaviors, including eating disorders and self-directed behaviors, however, varied in their occurrence within subjects across the periods. Most important, the significance of social companionship became obvious as the most severe forms of abnormal behavior, such as dissociative and self-injurious behaviors declined.

## 1. Introduction

Abnormal behavior, by definition, is “rarely seen in wild populations and does not promote the success and the survival of the individual or its close relatives” [1]. Abnormal behavior may be divided into qualitative abnormal behavior, which is not or only rarely found in wild populations, and quantitative abnormal behavior, which includes elements of normal behavior that are performed inappropriately in terms of context, sequence, frequency or duration [2]. It is well known that adverse early rearing conditions and restricted environments may be conducive to the development of abnormal behavior in diverse animal species, including humans [3,4]. Accordingly, reports on maladaptive behavioral abnormalities come almost exclusively from animals in captivity [4]. In the 1960s, Harlow and colleagues systematically induced behavioral deficiencies in non-human primates by isolating newborns from their mother (e.g., [5,6,7]). The maternally deprived infants developed invariant and repetitive stereotypic behavior patterns lacking any obvious function, supposedly caused by frustration, arousal and lack of stimulation (reviewed in [8,9]). Davenport’s [10] assumption, that in chimpanzees the development of stereotypic behavior may be prevented by maternal care during the first year of an infant’s life, proved wrong, as even mother-reared subjects developed abnormal behaviors in captive environments [11]. Furthermore, chimpanzees’ behavioral abnormalities persist into adulthood not only when maternally and peer deprived as very young infants, but also when deprived in their late infancy [12]. The first systematic documentation of abnormal behavior in rehabilitated chimpanzees was provided by Walsh *et al.* [13] and confirmed by, e.g., [14,15,16]. More recently, abnormal behaviors were also depicted quantitatively in zoo chimpanzees originating from various backgrounds [11,17]. These surveys revealed the impact of restricted rearing conditions on the development of specific abnormal behaviors [11,15]. The sooner the onset of maternal deprivation and the longer the lifetime spent in invasive research, the higher are the levels of behavioral aberrancies [16]. While different efforts, such as unpredictable feeding schedules [14], the provision of straw and forage material [18] and positive human interaction [19], were found to reduce the level of abnormal behaviors with oral components, such as coprophagy and regurgitation, these interventions did not ameliorate non-oral behavioral aberrancies, such as rocking and other stereotypies. On the other hand, it was noticed that coprophagy may spread by social transmission in groups of re-socialized chimpanzees [15,20,21]. 

Following Walsh *et al.* [13], abnormal behaviors of chimpanzees can be referred to as psychopathological, *i.e.*, as behavioral manifestations of psychological disorders, due to the social and psychobiological continuity between humans and great apes [22]. The susceptibility of primates and great apes in particular to psychological disorders, such as depression, post-traumatic stress disorder (PTSD), complex PTSD, generalized anxiety disorder and obsessive-compulsive disorders, was shown recently [23,24,25,26].

**Figure 1:**
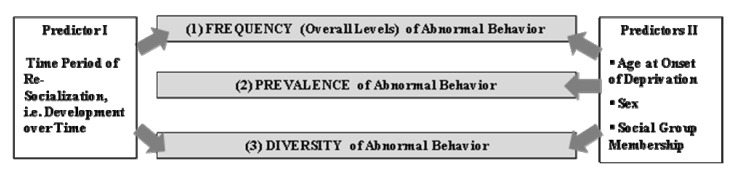
Levels of analysis.

In this study, we investigate individual variations of abnormal behaviors over a two-year period following re-socialization in a population of 18 adult former biomedical research chimpanzees. To our knowledge, no such long-term evaluation of the development of abnormal behavior in adult re-socialized chimpanzees has been undertaken yet. These chimpanzees had been single caged for around two decades and became members of one of three social groups in the course of a retirement project in Gaenserndorf, Austria in 2003. Beside group membership, the chimpanzees were classified as early (mean age of 1.2 years) or late deprived (mean age of 3.6 years) according to their age at arrival at the laboratory. Our first research question is dealing with the development of the overall levels and the diversity of abnormal behavior in the course of re-socialization (predictor I; see Figure 1). Due to the improvement of the physical and social environment, a decrease in the overall levels and the number of abnormal behaviors over the years might be expected. On the other hand, the abnormal behaviors may have become habits in these severely deprived chimpanzees [27,28] and, thus, be resistant to improved environments. This should be reflected in unvarying levels and a consistent number of abnormal behaviors over the years. Second, we analyzed the effects of the predictors I and II (see Figure 1) on the frequency, as well as on the diversity, of abnormal behavior simultaneously, *i.e.*, we investigated the development of the overall levels and the number of abnormal behaviors, respectively, of early and late deprived chimpanzees, males and females and the three social groups separately. The effects of age at onset of deprivation, sex and group membership on the overall levels, the prevalence and the diversity of abnormal behavior (predictors II; see Figure 1) were taken into account, because studies on mammals, including primates, found a negative relationship between the age at onset of infantile separation and the levels of abnormal behavior later on (reviewed in [4,16]). Sex was found to be a predictor for the development of abnormal behaviors in some studies [15,29], but not in others [17]. Differences between social groups may provide indications of social transmission of abnormal behaviors [21]. 

## 2. Results and Discussions

### 2.1. Frequency of Abnormal Behavior

#### 2.1.1. Development of Frequency over Time

In this study population, we observed 33 different abnormal behaviors and seven combinations of two behavior patterns (see Table 1). Across all 18 chimpanzees, the percent of scans spent on abnormal behavior did not differ significantly between sampling periods, *i.e.*, abnormal behavior was equally frequent in 2003, 2004 and 2005 in the social setting (see Table 2). The same was true when early (ED) and late deprived (LD) subjects, male and female chimpanzees and the AM group and the MS2 group (Table 2) were tested separately. In the MS1 group, by contrast, we found a significant increase in the performance of abnormal behaviors from 2003 to 2004 (Table 2).

**Table 1:** Ethogram of abnormal behavior patterns observed in this study population.

DISSOCIATIVE BEHAVIORS	
Floating limb ^7,9^	extended arm or leg is in convulsive movement
Stereotypic solitary play	invariant auto-play where one’s own leg or hands become(s) a play partner as if it/they do(es) not belong to the individual, often accompanied by play face
EATING DISORDERS	
Coprophagy & fecal manipulation ^1,2,3,4,5,6,9^	ingest and/or hold, carry or spread own feces on surface
Regurgitation ^1,2,3,4,6,9^	deliberate repetitive regurgitation; vomitus is retained within the mouth and re-ingested
REPETITIVE VOCALIZATIONS	
Gargling vocalization	sounds similar to gargling with water
Lip spluttering	splutter vocalization in non-grooming context, e.g., while resting
Raspberry vocalization ^1^	giving snuffling vocalizations, e.g., while auto-grooming
SELF-DIRECTED BEHAVIORS	
Anus poking ^9^	insert finger into own anus repetitively, then sometimes take a smell of finger
Ear poking ^6^	exaggerated, repetitive poking of one’s own finger into and pulling of own ear
Eye poking ^1,3,4,6,9^	poking of one’s own finger into own eye
Finger into mouth	repetitive pushing and pulling of finger into mouth rapidly
Finger nibbling and plucking	manipulate fingers with mouth and/or hand, affecting the skin
Fumble nipple ^3,8,9^	thumb or fingers are moved up and down rapidly to stimulate own nipple(s)
Groom pulled out hair	own pulled out hair is glued to the window and groomed
Hair pulling ^1,2,3,4,6,9^	pull out own hair with hand and/or mouth and ingest; usually during auto-grooming
Hand on ano-genital region	holding hand on ano-genital region, usually subsequent to self patting
Masturbation ^3,4,5^	masturbation directed at humans
Nail biting	chewing one`s own fingernails
Pat self	pat on body-parts with one or both hands
Self-clasp ^1,3,4,9^	clutch own shoulder or foot with knees pulled up to chest
Self-slap ^1,2^	slapping with palm at one’s own face or open mouth
*Stereotypic scratching	invariant repetitive scratching while sitting or lying
Suck hand ^1,4,5^	sucking one`s own hand
SELF-INJURIOUS BEHAVIOR	
*Bite self ^1,9^	bite in one’s own hand, accompanied by screaming
Excessive hair pulling	a bunch of hair is pulled out in non-grooming context
Hit self ^7,9^	hit own body-part with hand(s)
Self-mutilate ^1,4,5,7^	scratching at or grooming own body sufficient to cause injury
WHOLE-BODY STEREOTYPIES	
Bizarre postures ^1,2,3,4^	holding hand on head or into own mouth while sitting or moving in a sitting posture
Crouching and head waving	sitting in a crouched position and repeated moving of the head sideways
*Pacing ^2,3,9^	locomote quadrupedally in a circle with no clear objective
Rocking ^1,2,3,4,5,6,7,9^	sway repetitively and rhythmically bi- or quadrupedally from side-to-side, without piloerection
Stereotypic locomotion ^3^	one leg is moved in a repetitive exaggerated fashion while walking, leg motion is accompanied by audible exhalation
*Swagger and screaming	swagger with piloerection while single caged, accompanied by screaming
COMBINATIONS OF TWO (ABNORMAL ) BEHAVIORS	
Bite self and auto-grooming	self biting while auto-grooming
Hair pulling and anus poking	pull out hair with one hand and/or mouth and insert finger of the other hand into anus
Pat self and auto-grooming	self patting with one hand and auto-grooming with the other hand and/or mouth
Pat self and hair pulling	self patting with one hand and pull out hair with the other hand
Pat self and regurgitate	self patting while regurgitating
Pat self and self-clasp	self patting with one hand and self clasping with the other hand
Self-clasp and auto-grooming	self clasping with one hand and auto-grooming with the other hand and/or mouth

Abnormal behaviors were listed according to their occurrence in ^1 ^[13], ^2 ^[14], ^3 ^[16], ^4 ^[15], ^5 ^[11], ^6 ^[19], ^7 ^[24], ^8 ^[30] and ^9 ^[17]. * occurred only during single caging and were thus excluded from further analyses.

**Table 2:** Comparison of the mean percent of scans spent on overall abnormal behavior over the three sample periods (2003 to 2005).

	2003	2004	2005	
	Percent of Scans Mean ± SEM	Percent of Scans Mean ± SEM	Percent of Scans Mean ± SEM	Friedman Test; Dunn’s Post Test
**All **(N = 18)	10.7 ± 1.9	16.2 ± 2.9	15.0 ± 4.3	χ^2^ = 0.886, df = 2, *p* = 0.642
**ED **(N = 10)	12.7 ± 2.7	22.2 ± 3.4	20.7 ± 6.7	χ^2^ = 3.200, df = 2, *p* = 0.222
**LD **(N = 8)	8.1 ± 2.6	8.6 ± 3.7	7.8 ± 3.8	χ^2^ = 0.467, df = 2, *p* = 0.792
**M **(N = 10)	10.7 ± 3.3	16.6 ± 4.5	21.6 ± 7.1	χ^2^ = 2.205, df = 2, *p* = 0.332
**F **(N = 8)	10.7 ± 1.8	15.6 ± 3.8	6.7 ± 1.6	χ^2^ = 3.677, df = 2, *p* = 0.159
**AM **(N = 7)	14.3 ± 4.0	22.6 ± 4.7	29.0 ± 8.7	χ^2^ = 2.571, df = 2, *p* = 0.305
**MS1 **(N = 5)	9.4 ± 1.6	21.0 ± 3.8	8.0 ± 1.8	χ^2^ = 7.600, df = 2, *p* = 0.024;
				2003 *vs.* 2004: *p* < 0.05
**MS2** (N = 6)	7.5 ± 3.0	4.6 ± 2.5	4.4 ± 1.7	χ^2^ = 3.364, df = 2, *p* = 0.186

ED/LD = early/late deprived; M = male, F = female; AM = all-male, MS = mixed-sex.

#### 2.1.2. Effects of Age at Onset of Deprivation, Sex and Social Group Membership on the Frequency of Abnormal Behavior

**Figure 2:**
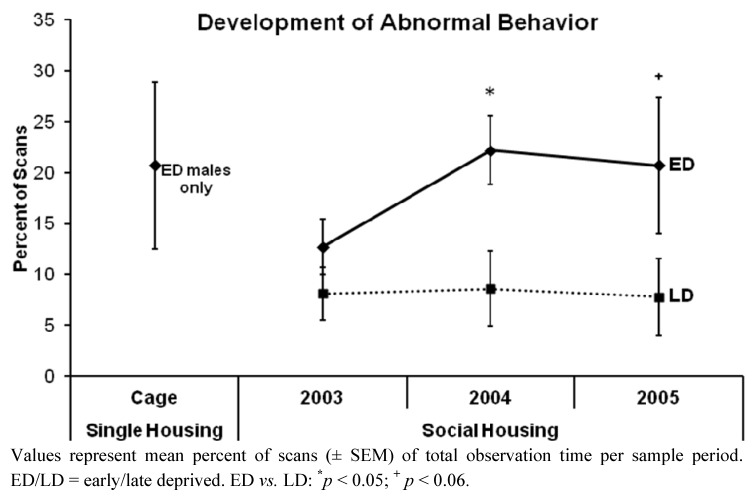
Development of the overall abnormal behavior of early *vs.* late deprived chimpanzees over the three sample periods.

When tested within each period (Figure 2), the percent of scans spent on overall abnormal behavior did not differ significantly between ED and LD chimpanzees in the period subsequent to re-socialization, *i.e.*, in 2003 (mean ± SEM: see Table 2; Mann-Whitney U Test: U = 24.500, *p* = 0.183). However, in the first year following re-socialization, *i.e.*, in 2004, the ED chimpanzees performed abnormal behaviors significantly more frequently than did LD ones (U = 15.000, *p* = 0.027), and in the second year following re-socialization, *i.e.*, in 2005, this trend was still recognizable (U = 18.000, *p* = 0.055), but not significant due to a higher variability among EDs. ED males did not differ in their mean percent of scans spent on overall abnormal behavior between the period of single caging (ED males: N = 6, mean ± SEM = 20.7 ± 8.2%) and subsequent to re-socialization, *i.e.*, in 2003 (mean ± SEM = 13.7 ± 4.6%; U = 14.000, *p* = 0.589; see Figure 2). The overall level of abnormal behavior of male and female chimpanzees did not differ, neither in 2003 (mean ± SEM: see Table 2; U = 36.000, *p* = 0.762) and 2004 (U = 40.000, *p* = 0.965), nor in 2005 (U = 27.500, *p* = 0.286). In 2003, as well as in 2005, we found no difference in the percentage of scans spent on overall abnormal behavior between the three social groups (mean ± SEM: see Table 2; 2003: Kruskal-Wallis Test: H = 2.088, *p* = 0.352; 2005: H = 5.889, *p* = 0.053). However, in the first year following re-socialization, *i.e.*, in 2004, the AM group significantly exceeded the MS2 group in their performance of abnormal behaviors (H = 7.940, *p* = 0.019, Dunn’s Post Test: AM *vs.* MS2: *p* < 0.05).

### 2.2. Prevalence of Abnormal Behavioral Categories and Specific Abnormal Behaviors

We did not differentiate between the three sample periods when analyzing the proportion of individuals who showed an abnormal behavior pattern in the social setting. Altogether, 29 different abnormal behaviors and seven combinations of two (abnormal) behaviors were detected between 2003 and 2005 (see Table 3). While more than half of the observed abnormal behaviors (56%, *i.e.*, 20 out of 36) were highly individual, 44% (*i.e.*, 16 out of 36) were shown by more than one individual (range: 2–13 individuals). The median percent of scans per abnormal behavior was highly variable and ranged from 0.2% to 9.1% of scans. The range within specific abnormal behavior patterns was even more variable and was most extreme in “Regurgitation”, where it varied between 0.5 and 41.8% of scans(see Table 3).

**Table 3:** Prevalence of abnormal behavior patterns, expressed as the proportion of individuals who performed a specific abnormal behavior pattern relating to the total number of study subjects.

	Prevalence	Median	Range
Behavior Pattern	Percent (No. of Ind.)	Percent of Scans	Percent of Scans
DISSOCIATIVE BEHAVIORS			
Stereotypic solitary play	17 (3)	0.9	0.1–1.0
Floating limb	6 (1)	0.2	
EATING DISORDERS			
Coprophagy & fecal manipulation	72 (13)	1.0	0.3–2.9
Regurgitation	39 (7)	5.4	0.5–41.8
REPETITIVE VOCALIZATIONS			
Lip spluttering	28 (5)	2.0	0.5–9.2
Raspberry vocalization	6 (1)	0.4	
Gargling vocalization	6 (1)	0.2	
SELF-DIRECTED BEHAVIORS			
Hair pulling	67 (12)	1.7	0.1–12.1
Pat self	61 (11)	0.8	0.2–9.6
Hand on ano-genital region	44 (8)	1.0	0.2–1.4
Self-clasp	22 (4)	0.4	0.3–0.8
Fumble nipple	17 (3)	0.4	0.2–0.6
Self-slap	11 (2)	0.3	0.3
Masturbation	11 (2)	0.4	0.2–0.6
Finger nibbling and plucking	6 (1)	4.4	
Groom pulled out hair	6 (1)	0.2	
Finger into mouth	6 (1)	1.0	
Nail biting	6 (1)	5.7	
Suck hand	6 (1)	1.2	
Ear poking	6 (1)	0.2	
Eye poking	6 (1)	0.3	
Anus poking	6 (1)	0.6	
SELF-INJURIOUS BEHAVIOR			
Hit self	11 (2)	1.9	0.2–3.6
Self-mutilate	6 (1)	9.1	
Excessive hair pulling	6 (1)	0.2	
WHOLE-BODY STEREOTYPIES			
Bizarre postures	11 (2)	0.2	0.2
Rocking	6 (1)	0.3	
Stereotypic locomotion	6 (1)	2.1	
Crouching and head waving	6 (1)	0.2	
COMBINATIONS OF TWO (ABNORMAL) BEHAVIORS			
Pat self & auto-grooming	28 (5)	1.7	0.2–7.0
Pat self & hair pulling	17 (3)	1.0	0.2–2.1
Self-clasp & auto-grooming	11 (2)	0.2	0.1–0.2
Pat self & self-clasp	6 (1)	0.2	
Pat self & regurgitate	6 (1)	0.2	
Bite self & auto-grooming	6 (1)	0.2	
Hair pulling & anus poking	6 (1)	0.2	

#### 2.2.1. Effects of Age at Onset of Deprivation, Sex and Social Group Membership on the Prevalence of Abnormal Behavioral Categories and Specific Abnormal Behaviors

The impact of the different predictors could only be tested for the two most prevalent abnormal behavioral categories “Eating Disorders” and “Self-directed Behaviors”. “Eating Disorders” were significantly more prevalent in LDs than in EDs (100% *vs.* 70%, Chi-Square Test: χ^2^ = 5.294, df = 1, *p* = 0.021) and in the two mixed-sex groups compared to the AM group (AM *vs.* MS1 *vs.* MS2: 57% *vs.* 100% *vs.* 100%, χ^2^ = 14.389, df = 2, *p* = 0.001). It did not differ significantly between male and female individuals (70% *vs.* 88%, χ^2^ = 2.051, df = 1, *p* = 0.152). There was a trend for “Self-directed Behaviors” to be more prevalent in EDs compared to LDs (100% *vs.* 75%, χ^2^ = 3.571, df = 1, *p* = 0.059). “Self-directed Behaviors” were significantly more widespread in the AM and MS1 group compared to the MS2 group (AM *vs.* MS1 *vs.* MS2: 100% *vs.* 100% *vs.* 67%, χ^2^ = 8.157, df = 2, *p* = 0.017). It did not differ significantly between male and female chimpanzees (90% *vs.* 88%, χ^2^ = 0.022, df = 1, *p* = 0.881). 

The comparison of the three most prevalent abnormal behaviors “Coprophagy & fecal manipulation”, “Hair pulling” and “Pat self” between ED and LD chimpanzees revealed that “Coprophagy & fecal manipulation” was significantly more prevalent among LD than ED chimpanzees (88% *vs.* 60%, Chi-Square Test: χ^2^ = 5.297, df = 1, *p* = 0.026), in the two mixed-sex groups compared to the AM group (AM *vs.* MS1 *vs.* MS2: 29% *vs.* 100% *vs.* 100%, χ^2^ = 44.026, df = 2, *p* < 0.001) and among females than males (100% *vs.* 50%, χ^2^ = 16.667, df = 1, *p* < 0.001). “Pat self”, by contrast, was significantly more prevalent in ED compared to LD chimpanzees (90% *vs.* 25%, χ^2^ = 36.739, df = 1, *p* < 0.001). We found “Pat self” to be more widespread in the AM and the MS1 group compared to the MS2 group (AM *vs.* MS1 *vs.* MS2: 86% *vs.* 80% *vs.* 17%, χ^2^ = 47.902, df = 2, *p* < 0.001). It did not differ between males and females (60% *vs.* 63%, χ^2^ = 0.073, df = 1, *p* = 0.857). “Hair pulling” did not differ between the ED and LD subjects (70% *vs.* 63%, χ^2^ = 0.368, df = 1, *p* = 0.603) and between males and females (70% *vs.* 63%, χ^2^ = 0.368, df = 1, *p* = 0.603. However, “Hair pulling” was significantly more widespread in the AM and the MS1 group compared to the MS2 group (AM *vs.* MS1 *vs.* MS2: 71% *vs.* 80% *vs.* 50%, χ^2^ = 7.075, df = 2, *p* = 0.030). 

#### 2.2.2. Persistency of the Most Prevalent Abnormal Behavioral Categories and Specific Abnormal Behaviors

“Eating Disorders” varied substantially within subjects across the three sample periods (Kendall’s Rank Correlation: 2003 *vs.* 2004: τ = 0.161, *p* = 0.506; 2003 *vs.* 2005: τ = 0.204, *p* = 0.401; 2004 *vs.* 2005: τ = 0.316, *p* = 0.192). We found no correlation in the occurrence of “Self-directed Behaviors” of individual chimpanzees between 2003 and 2004 (τ = 0.396, *p* = 0.103), as well as between 2003 and 2005 (τ = 0.255, *p* = 0.293). Between 2004 and 2005, however, the occurrence of “Self-directed Behaviors” within subjects stabilized (τ = 0.837, *p* = 0.001).

We found the occurrence of “Coprophagy” to be highly variable within subjects across the three sample periods (2003 *vs.* 2004: τ = 0.125, *p* = 0.606; 2003 *vs.* 2005: τ = 0.204, *p* = 0.401; 2004 *vs.* 2005: τ = 0.255, *p* = 0.293). We found no correlation in the occurrence of “Pat self” of individual chimpanzees between 2003 and 2004 (τ = 0.396, *p* = 0.103), as well as between 2003 and 2005 (τ = 0.255, *p* = 0.293) due to an increase in the number of chimpanzees performing the respective behavior (see Table S1). Between 2004 and 2005, however, spreading of “Pat self” within subjects did not change (τ = 0.671, *p* = 0.006). The occurrence of “Hair pulling” within subjects remained more or less constant between 2003 and 2004 (τ = 0.553, *p* = 0.023), but varied between 2003 and 2005 (τ = 0.236, *p* = 0.331), as well as between 2004 and 2005 (τ = 0.224, *p *= 0.357).

### 2.3. Diversity of Abnormal Behaviors

All 18 chimpanzees of our study population exhibited abnormal behaviors. The number of different abnormal behaviors per individual varied between one and 12 (see Table 4a). However, the number of persistent abnormal behaviors per individual, *i.e.*, behaviors that occurred at all three sample periods, ranged between zero and two, except for one female (see Table 4b).

Table 4**(a)** Diversity of abnormal behaviors, *i.e.*, the total number of different abnormal behaviors per individual shown between 2003 and 2005. **(b)** Number of persistent abnormal behaviors per individual, *i.e.*, the number of abnormal behaviors that occurred at all three sample periods.

**(a):** 

Individual	Deprivation Class	Sex	Age in 2003 (in Years)	No. of Abnormal Behaviors
ALL-MALE GROUP (= AM)				
Blacky	ED	M	18	6 (11)
Gogo	ED	M	29	5 (6)
Isidor	ED	M	25	9 (9)
Jakob	LD	M	20	6
Johannes	ED	M	22	7 (9)
Max	ED	M	25	5 (5)
Michi	ED	M	22	5 (7)
MIXED-SEX GROUP 1 (= MS1)				
Clyde	LD	M	21	5
Gabi	ED	F	24	7
Ingrid	ED	F	24	8
Martha	ED	F	25	11
Pünktchen	LD	F	20	12
MIXED-SEX GROUP 2 (= MS2)				
Anton	LD	M	21	2
Bonnie	LD	F	21	5
Helene	LD	F	21	1
Moritz	LD	M	20	1
Schuscha	LD	F	21	5
Susi	ED	F	29	4

**(b):** 

Individual	No. of Persistent Abnormal Behaviors	Persistent Abnormal Behaviors	% of Scans 2003	% of Scans2004	% of Scans 2005
Blacky	0(0)	-	-	-	-
Gogo	1(1)	Stereotypic locomotion	3.1	0.5	2.2
Isidor	0(0)	-	-	-	-
Jakob	1	Regurgitation	17.0	20.4	31.6
Johannes	1(1)	Pat self	4.5	6.2	14.7
Max	1(1)	Regurgitation	28.1	41.9	70.6
Michi	2(1)	Pat self Hair pulling	0.7 0.7	4.6 23.1	2.9 20.6
Clyde	1	Self-slap	3.8	4.0	3.0
Gabi	2	Pat self Pat self & Autogrooming	5.3 2.8	5.5 9.6	6.5 6.9
Ingrid	1	Pat self	8.2	17.4	6.0
Martha	4	Pat self Regurgitation Coprophagy Stereotypic solitary play	5.0 1.8 5.4 1.1	2.7 4.0 1.3 1.3	2.2 0.9 0.9 0.4
Pünktchen	2	Hair pulling Regurgitation	4.5 0.7	2.9 0.7	0.4 1.3
Anton	0	-	-	-	-
Bonnie	1	Lip spluttering	0.4	0.7	0.4
Helene	0	-	-	-	-
Moritz	0	-	-	-	-
Schuscha	2	Hair pulling Coprophagy	12.5 1.1	0.3 2.4	5.7 1.2
Susi	1	Lip spluttering	12.0	12.5	2.0

ED/LD = early/late deprived; M = male, F = female. In parenthesis: including abnormal behaviors performed during single caging.

#### 2.3.1. Development of Diversity over Time

If we differentiate between the three sample periods, the 18 subjects of our study population did not differ significantly with respect to their diversity of abnormal behaviors (see Table 5). The same was true for ED and LD chimpanzees and for male and female subjects (see Table 5). The AM and the MS2 group did not differ in their diversity of abnormal behaviors over the years, whereas the MS1 group exhibited a significantly higher number of abnormal behaviors in the first year compared to the second year following re-socialization (Table 5). For the AM group, we ran a second analysis where we also included the diversity of abnormal behaviors during single caging (cage: mean ± SEM = 3.9 ± 0.9). However, no significant difference between the four activity periods became apparent (Friedman Test: χ^2^ = 1.286, df = 2, *p* = 0.733). 

**Table 5:** Comparison of the diversity, *i.e.*, the mean number of abnormal behaviors, over the three sample periods (2003 to 2005).

	2003	2004	2005	Friedman Test;
	No. of Abnormal	No. of Abnormal	No. of Abnormal	Dunn’s Post Test
	Behaviors	Behaviors	Behaviors	
	Mean ± SEM	Mean ± SEM	Mean ± SEM	
**All** ( N = 18)	2.8 ± 0.4	4.3 ± 3.5	2.8 ± 0.4	χ^2^ = 1.909, df = 2, *p* = 0.385
**ED** (N = 10)	3.1 ± 0.4	5.0 ± 1.0	3.2 ± 0.6	χ^2^ = 2.811, df = 2, *p* = 0.245
**LD** (N = 8)	2.5 ± 0.6	3.4 ± 1.4	2.4 ± 0.5	χ^2^ = 0.069, df = 2, *p* = 0.966
**M** (N = 10)	2.4 ± 0.4	3.0 ± 0.7	2.5 ± 0.5	χ^2^ = 0.722, df = 2, *p* = 0.697
**F** (N = 8)	3.4 ± 0.6	5.9 ± 1.5	3.3 ± 0.6	χ^2^ = 1.867, df = 2, *p* = 0.393
**AM** (N = 7)	2.9 ± 0.9	3.7 ± 0.7	2.9 ± 0.7	χ^2^ = 1.280, df = 2, *p* = 0.527
**MS1** (N = 5)	3.8 ± 0.6	8.4 ± 1.4	3.6 ± 0.9	χ^2^ = 7.600, df = 2, *p* = 0.024;
				2004 *vs.* 2005: *p* < 0.05
**MS2** (N = 6)	2.0 ± 0.7	1.5 ± 0.7	2.2 ± 0.6	χ^2^ = 1.810, df = 2, *p* = 0.405

ED/LD = early/late deprived; M = male, F = female; AM = all-male, MS = mixed-sex.

#### 2.3.2. Effects of Age at Onset of Deprivation, Sex and Social Group Membership on the Diversity of Abnormal Behaviors

ED *vs.* LD individuals did not differ with respect to the diversity of abnormal behaviors in the social setting (mean ± SEM: see Table 5; Mann-Whitney U Test: 2003: U = 29.500, *p* = 0.372; 2004: U = 27.000, *p* = 0.266; 2005: U = 33.500, *p* = 0.593). Likewise, we found no difference between males and females (mean ± SEM: see Table 5; 2003: U = 26.000, *p* = 0.229; 2004: U = 24.000, *p* = 0.168; 2005: U = 30.000, *p* = 0.397). The three social groups did not differ in their diversity of abnormal behaviors in 2003 and in 2005 (mean ± SEM: see Table 5; Kruskal-Wallis Test and Dunn’s Post Test: 2003: H = 4.492, *p* = 0.106; 2005: H = 1.209, *p* = 0.567). However, in 2004, individuals of the MS1 group significantly exceeded those of the MS2 group concerning the number of abnormal behaviors (mean ± SEM: see Table 5; H = 10.013, *p* = 0.007, Dunn’s Post Test: MS1 *vs.* MS2: *p* < 0.01).

### 2.4. Discussions

#### 2.4.1. Overall Abnormal Behavior

Since the levels of abnormal behavior did not change significantly over the two-year period, neither within the whole study population, nor within early- or late-deprived subjects, males or females and within two out of three social groups, it might be suggested that an emancipation from original causes [27] took place in most of the abnormal behaviors performed by our study population. However, more detailed analyses revealed that the individual performance of the most prevalent abnormal behaviors varied substantially across the three sample periods. Thus, it seems that most of these abnormal behaviors are malleable. In other words, although the time spent with abnormal behavior was stable, the composition of abnormal behaviors did vary. As we cannot rule out that some short-lasting abnormal behaviors may have been missed by conducting scan sampling, we will clarify this question in detail according to Mason & Latham’s [28] classification in an ongoing study based on focal sampling data recorded in 2004. 

Unexpectedly, in one of the two mixed-sex groups, the level of abnormal behavior even increased in the first year following re-socialization. Social group formation may cause an increase in the level of abnormal behaviors, at least in the short-run [20], since individuals have to adapt to the unfamiliar social stimulation. However, mean values of abnormal behaviors returned to the initial level in the second year of group-living. At least for the males of our study population, it could be demonstrated that elevated stress levels did not contribute to these increasing levels of abnormal behavior, since the fecal cortisol metabolite levels decreased during the early re-socialization period compared to the levels found during the habituation period [31]. Thus, it can be assumed that our study subjects are able to cope with social stressors. Nevertheless, a contrary effect became obvious within the ED individuals whose mean levels of abnormal behavior decreased subsequent to re-socialization, but one year after successful social rehabilitation, they increased again to values comparable to that found in the ED males during single caging and stayed at this high level throughout the second year of group-living. LD individuals differed significantly from EDs within these two years after re-socialization, which is in line with Warniment and Brent [16], who report of significant effects of age at onset of social deprivation on the level of abnormal behavior. Similar to the return of our re-socialized ED chimpanzees to the original levels exhibited during single caging, Baker [32] found no difference in the level of abnormal behavior between single and pair- or trio-housed chimpanzees after living in these settings for years, though the level in these biomedical chimpanzees lay in between that of our ED and LD individuals. Moreover, while ED chimpanzees spent about five percent of scans on affiliative social behavior subsequent to re-socialization, this level halved in the first and second year of group-living (Kalcher-Sommersguter, unpublished data). Accordingly, we assume that the abnormal behavior may have been replaced to some extent by social activity in ED chimpanzees in the short-run, but afterwards returned to their pattern of “non-social” abnormal behavior. LD chimpanzees, by comparison, invariably spent about eleven percent of their time on affiliative behavior (Kalcher-Sommersguter, unpublished data). Fritz and Howell [33] observed a decrease in affiliative interactions in rehabilitated chimpanzees as well and, as a solution, proposed to vary the composition of social groups after one to three years to stimulate social activity again. Sex did not affect the level of abnormal behavior per sample period, just as reported by Birkett and Newton-Fisher [17], whereas effects of group composition became apparent, as the ED-dominated all-male group significantly exceeded the LD-dominated mixed-sex group 2 in the first year following re-socialization. 

#### 2.4.2. Prevalence and Diversity of Abnormal Behaviors

We found 29 different abnormal behavior patterns that occurred in the social setting, which is virtually equal to the 26 different behaviors found by Walsh *et al.* [13], even though only half of the observed abnormal behaviors were identical between these two studies. Just as in zoo-living chimpanzees [17], coprophagy and hair pulling were most prevalent in this population of ex-laboratory chimpanzees. With respect to coprophagy, even median percent of time and scans spent on this behavior, respectively, are virtually identical, whereas our chimpanzees spent much more time on regurgitation compared to the zoo-living individuals [17]. We did not distinguish between eating or manipulating the feces, as these patterns mostly occurred in combination in our study population.

Eating disorders, including coprophagy and regurgitation, are presumed to be associated with the feeding regime in captivity [34], such as insufficient dietary roughage and lack of foraging opportunities [35], as well as with social deprivation and lack of adequate stimulation [13]. In our study population, eating disorders, mainly in terms of coprophagy, were significantly more prevalent in the LD compared to ED chimpanzees. Nash *et al.* [15] found coprophagy to be more widespread among mother-reared chimpanzees compared to hand-reared ones and suggested coprophagy to spread by social learning. We did not observe social transmission of coprophagy across the three sample periods, however, the longer time period of early social housing of LDs might at least partially explain this outcome. Unfortunately, reliable data on the prevalence of eating disorders, including coprophagy and regurgitation, during the laboratory setting are not available. However, two cases of social transmission of regurgitation occurred. In the first case, the female, Bonnie, was housed together with the male, Max, because both participated in a breeding program in the late 1990s. This co-housing had to be stopped shortly after, since Bonnie started to regurgitate as did Max [36]. In the second case, the male, Blacky, who had been regurgitating already during single caging, was observed to accurately reproduce the sequence and mode of regurgitation of the male, Max, during social housing in 2004 [37]. Moreover, in the social setting, regurgitation was found to be persistent in four out of seven individuals who performed this behavior. That regurgitation is extremely difficult to overcome was shown in a case study where even behavioral training along with a dietary change could not eliminate this unhealthy behavior [38], possibly due its presumed self-stimulatory and comforting effect (reviewed in [39]). However, while the levels were stable or even decreased in the four females, they increased in the three male chimpanzees. Thus, it can be assumed that these males met the criteria for obsessive-compulsive disorder [26]. 

Self-directed behaviors, including self-patting and hair pulling, contrary to eating disorders, were more widespread among ED compared to LD subjects. All ED chimpanzees exhibited self-patting similar to most human infants reared in restricted environments and lacking maternal stimulation, who perform this self-stimulating abnormal behavior as well [40]. Beyond that, self-patting became more widespread in the whole study population in the two years following re-socialization, possibly by social transmission. Hair pulling is triggered by long-term exposure to anxiety- and boredom-inducing environments in diverse animal species, from mice to non-human and human primates (reviewed in [41]). Accordingly, hair pulling was the most prevalent self-directed behavior in our study population and did not differ between ED and LD chimpanzees, as all of them spent decades in similar adverse environments. Self-clasping, which was more common in our study population than in zoo-living chimpanzees [17], is associated with social deprivation, as it emerges when clinging to the mother is unachievable and, thus, has to be redirected to the own body (reviewed in [42]). Furthermore, the occurrence of self-clasping might provide an indication of generalized anxiety disorder (GAD) in chimpanzees [26]. It occurred in three of four ED females, but in none of our ED males, which gives rise to the suspicion that females might be more vulnerable to the absence of body contact in early infancy and consequently to the development of GAD, which is in line with findings from humans [43].

More severe categories of abnormal behaviors, such as dissociative behaviors, whole-body stereotypies and self-injurious behavior (SIB) were rare in our study population. Three chimpanzees in our study population exhibited dissociative behaviors—in humans associated with traumatic life events [44]—where, e.g., a dissociated limb served as a play partner or a limb was “floating”. Whole-body stereotypies, which are mainly caused by social deprivation [45], disappeared in two of four subjects after the period subsequent to re-socialization, similarly to what Fisher *et al.* [46] found in institutionalized children years after adoption. That rocking was extremely rare in our chimpanzees might be due to the fact that the cages were freely suspended and swinging in the first years in the laboratory and, thus, seemed to provide the respective stimulation [47]. Additionally, low levels of rocking might also be traced back to the fact that these chimpanzees experienced at least some social companionship in early infancy, and rocking was found to be most prevalent in subjects who were separated from their mother within the first months of life [48]. Adverse early rearing conditions, repeated biomedical experimentation and single housing are supposed to be the main factors linked to the etiology of SIB in non-human primates, which in turn is assumed to serve affect-regulatory functions as it does in humans [49]. SIB occurred only once and was ceased, respectively, in two ED males in the social setting. Briefly summarized, in six out of seven chimpanzees who performed severe abnormal behaviors, the behaviors disappeared in the course of group-living. Thus, altogether, an improvement in the most severe categories of abnormal behavior is recognizable, emphasizing the curative potential of social companionship. Further evidence of social housing serving a therapeutic role is provided by the fact that stereotypic scratching, pacing and swagger and screaming were not observed at all when the chimpanzees were socially housed. Table S1 provides an overview of the occurrence of all seven abnormal behavioral categories per individual and year in the social setting.

Concordant to the findings of Birkett and Newton-Fisher [17], all study subjects exhibited abnormal behaviors with a similar range with respect to the individual diversity (1–12 *vs.* 2–14), but while all our ED subjects exhibited at least four different abnormal behaviors, LD individuals displayed a broader distribution. However, the overall number of different abnormal behaviors was not affected by any of the predictors, except for group composition. This was caused by the two females in the MS1 group—Martha and Pünktchen—who exhibited the highest variety of abnormal behaviors in this study population. Focusing on the persistence of abnormal behaviors revealed that only a small fraction of the abnormal behaviors occurred at all three sample periods, *i.e.*, solely eight out of a total of 33 different abnormal behaviors. Almost half of the study population exhibited one persistent abnormal behavior. These persistent abnormal behaviors may be so-called self-stimulatory behaviors defined as “learned, operant behaviors for which the reinforcers are the perceptual stimuli automatically produced by the behavior” and which are extremely difficult to eliminate, as the reinforcing properties are also retained in enriched environments [50].

Our study had methodological weaknesses, as we conducted scan sampling and, thus, could not evaluate the absolute frequencies and durations of specific abnormal behaviors. Furthermore, as some rarely occurring abnormal behaviors with short durations may have been missed, results have to be interpreted with caution. Additionally, as our sample size was modest (N = 18), the results may not generalize to a larger population of captive chimpanzees. The strength of this study, however, is the expanded observation time over a period of two years, which gives insight into the development of abnormal behaviors of rehabilitated chimpanzees in the course of group-living.

## 3. Methods

### 3.1. Ethics Statement

This study was carried out in accordance with the recommendations of the US National Research Council [51] and with the Austrian Federal Act on the Protection of Animals. The retirement process was recommended by a board of experts, including J.A.R.A.M. van Hooff (Emeritus University of Utrecht), Mike Seres (formerly MPI Leipzig), Janet Gonder (formerly Baxter) and Joerg Eichmann (then consultant to Baxter) and conducted under the direction of Signe Preuschoft. 

### 3.2. Subjects and Housing

The study subjects were 18 adult chimpanzees (10 males and 8 females, aged between 18 and 29 years at the onset of this study; see Table 4a), who were imported from Africa to the Austrian Immuno Laboratories between 1976 and 1986. According to their estimated age upon arrival at the laboratory, the chimpanzees could be assigned to two different deprivation classes: the chimpanzees imported between 1976 and 1982 arrived as very young infants (mean age of 1.2 ± 0.4 years) and were socially deprived upon arrival; the chimpanzee orphans arriving in 1986 were in their later infancy (mean age of 3.6 ± 0.5 years) and spent the first year as a peer group before being single caged. Accordingly, we classified the former as “early deprived” (ED, N = 10) and the latter as “late deprived” (LD, N = 8). The only exception was the male, Blacky, who arrived in 1986 at an estimated age of one year and was socially deprived immediately upon arrival; thus, we categorized him as an early deprived chimpanzee. All of these chimpanzees had undergone severe deprivation as they spent between 16 and 27 years without physical contact to conspecifics. The chimpanzees were part of research protocols, mainly on Hepatitis and HIV. From these experiments, our 18 study subjects emerged as uninfected. In December 2002, the chimpanzees were relocated to their “retirement home”—the newly constructed primate indoor facility in Gaenserndorf, Austria—designed for group housing. In spring 2003, the re-socialization project was initialized, which resulted in the formation of three social groups by October 2003: an all-male group of seven adult individuals (AM), a one-male mixed-sex group of five adults (MS1) and a two-male mixed-sex group of 6 adult and 3 juvenile chimpanzees (MS2). The three social groups inhabited separate indoor enclosures of 10 × 13 × 6 m (MS1 group) and 16 × 13 × 6 m (AM and MS2 group) during daytime. The male chimpanzees spent the night in their single cages of 2 × 3 × 3 m furnished with hammocks and straw for bedding, and the females spent the night in pairs or with their juvenile offspring in two interconnected cages. The chimpanzees were fed four times a day: in the morning in the night cages, at mid-day and early afternoon in the indoor enclosures and in the evening in the night cages again. Additionally, foraging material was provided frequently. The feeding regime did not change during the re-socialization period. For more details on the study population, see Kalcher *et al.* [12]. 

### 3.3. Data Collection

In the first weeks after the chimpanzees’ arrival at the primate house in Gaenserndorf, the chimpanzees remained in their night cages to get habituated to the new environment. We conducted video recordings of ED males (N = 6) during this period of single housing in January and February 2003 and analyzed their abnormal behavior for the purpose of comparison between single and group housing. The analyses of abnormal behavior during single housing are based on a total of 180 hours of 5-minute scan sampling [52], which corresponds to 30 hours or 363 scans per individual evenly distributed between 8 a.m. and 6 p.m. 

Furthermore, we collected data on abnormal behavior of all 18 chimpanzees in the social setting between (1) October 2003 and January 2004 (the period subsequent to re-socialization), (2) February and July 2004 (the first year following re-socialization) and (3) February and June 2005 (the second year following re-socialization). Sampling was distributed evenly over the chimpanzees’ activity period while they were in the indoor enclosures, that is, between 9 a.m. and 5 p.m. in 2003 and 2004 (except for members of the AM group, who returned in 2004 to their night cages at 2.25 p.m. due to management reasons). In 2005, the social activity period, *i.e.*, the time the individuals spent together in the indoor enclosure, varied between the three groups: 9 a.m. to 12.30 a.m. for the AM group, 9 a.m. to 3.30 p.m. for the MS1 group and 9 a.m. to 4 p.m. for the MS2 group, due to financial constraints. Results on abnormal behavior in the social setting are based on 187.5 hours (72 h in 2003, 64.5 h in 2004 and 51 h in 2005) of 5-minute scan sampling. The overall number of scans per individual during the period of social setting ranges from 612 (AM group) to 825 (MS2 group). 

Specific abnormal behaviors were assigned to seven categories: Dissociative Behaviors according to [24], Eating Disorders according to [44] (comparable to Food- and Feces-Related Behaviors in [16] and Appetitive Disorders in [34]), Repetitive Vocalizations and Self-Directed Behaviors according to [16], Self-Injurious Behaviors and Whole-Body Stereotypies according to [53] and Combinations of Two (Abnormal) Behaviors. The abnormal behaviors shown over the three (ED females and all LDs) and four sample periods (ED males), respectively, are listed in Table 1. To allow comparability to other studies, we refrained from subsuming rarely occurring abnormal behaviors as other abnormal behaviors and defined each abnormal behavior separately. 

### 3.4. Data Analysis

We used three dependent variables. First, we evaluated the frequency with which any individual exhibited abnormal behavior, expressed as the percentage of scans during which abnormal behavior was performed. Second, we calculated the prevalence of a specific behavioral category as the proportion of individuals who performed that aberrancy out of the total number of subjects. Third, we assessed the diversity of aberrancies per individual by counting the number of different abnormal behaviors performed. Note that for the MS2 group, only the six adult individuals were considered in the analyses. To enable comparisons with other studies, e.g., Birkett & Newton-Fisher [17], for the variable prevalence median percent of scans and range is shown. Data on abnormal behavior were normally distributed, therefore for the variables frequency and diversity mean values ± SEM are shown. Predictors were the time period of re-socialization (2003, 2004, 2005), age at onset of deprivation (ED *vs.* LD), sex and social group membership (AM, MS1, MS2).

Due to the small sample size, we conducted nonparametric tests, as they are more robust regarding outliers than their parametric pendants [54]. Friedman Tests were conducted to investigate the effect of the time period of re-socialization on the frequency and diversity of abnormal behaviors. To identify the effects of age at onset of deprivation and sex on the frequency and diversity of abnormal behaviors per sampling period, Mann-Whitney U Tests were conducted. We also compared frequency and diversity of abnormal behavior between the three social groups using Kruskal-Wallis Tests and Dunn’s Post Tests. We performed Chi-Square Tests in order to test for effects of age at onset of deprivation, sex and social group membership on the prevalence of abnormal behavioral categories and specific abnormal behaviors. To evaluate the individual consistency of the most prevalent abnormal behavioral categories and specific abnormal behaviors over the three sample periods, we conducted Kendall’s Rank Correlations. Analyses were conducted in GraphPad InStat and SPSS version 16.0. Mann-Whitney U Tests were two-tailed and the alpha level was set at 0.05 for all tests. *p*-values between 0.051 and 0.059 are reported as a trend.

## 4. Conclusions

The pervasive effects of severe and long-term deprivation became apparent, as abnormal behaviors were present in the entire study population, and the mean level of the overall abnormal behavior clearly surpassed the levels found in other populations [11,14,17]. Moreover, consistent with our findings with respect to social behavior [12,55,56], age at onset of infantile deprivation had an impact on the overall levels of behavioral aberrancies as well. Except for coprophagy, which was more widespread among females compared to males, no sex differences were found. The overall levels of abnormal behavior remained stable over the three sample periods, though the composition of these behaviors per individual varied substantially. Most important, the improvement in the most severe types of abnormal behaviors revealed the therapeutic role of social companionship in overcoming psychological disorders, at least to some extent. 

## Supplementary

**Table S1:** Prevalence of the abnormal behavioral categories per individual and year.

		Eating Disorders			Dissociative Behaviors			Repetitive Vocalizations			Self-directed Behaviors			Self-injurious Behaviors			Whole Body Stereotypies			Combinations of two Behaviors		
		2003	2004	2005	2003	2004	2005	2003	2004	2005	2003	2004	2005	2003	2004	2005	2003	2004	2005	2003	2004	2005
AM GROUP	Blacky		x	x					x		x			x								
	Gogo		x		x	x						x	x				x	x	x			
	Isidor							x	x		x	x	x				x				x	
	**Jakob**	**x**	**x**	**x**							**x**	**x**	**x**								**x**	
	Johannes										x	x	x			x	x					x
	Max	x	x	x				x				x	x									
	Michi										x	x	x									
MS1 GROUP	**Clyde**		**x**	**x**							**x**	**x**	**x**									
	*Gabi*	*x*	*x*								*x*	*x*	*x*							*x*	*x*	*x*
	*Ingrid*	*x*	*x*								*x*	*x*	*x*								*x*	*x*
	*Martha*	*x*	*x*	*x*	*x*	*x*	*x*				*x*	*x*	*x*								*x*	*x*
	***Pünktchen***	***x***	***x***	***x***							***x***	***x***	***x***				***x***	***x***		***x***	***x***	
MS2 GROUP	**Anton**	**x**		**x**									**x**									
	***Bonnie***	***x***	***x***					***x***	***x***	***x***		***x***	***x***									
	***Helene***	***x***		***x***																		
	**Moritz**	**x**																				
	***Schuscha***	***x***	***x***	***x***	***x***			***x***		***x***	***x***	***x***	***x***									
	*Susi*		*x*	*x*				*x*	*x*	*x*		*x*	*x*									

Names: standard font = early deprived, bold = late deprived, italics = females.
